# The Indian fashion and textile sector in and post COVID-19 times

**DOI:** 10.1186/s40691-021-00267-4

**Published:** 2022-05-05

**Authors:** Karan Khurana

**Affiliations:** Webster University, Tashkent, Uzbekistan

**Keywords:** COVID-19, Fashion industry, Digitization, Value chain analysis, Developing economies

## Abstract

The fashion and textile sectors have proved to be the socio-economic booster for developing countries in the last two decades. This article looks into the challenges faced by the Indian fashion and textiles sector in and post-pandemic. The current COVID-19 crisis has presented the sector with a unique set of challenges that are indeed the future strategies.

Primary and secondary research methods were used to explore the impact of the pandemic on the sector in India. A systematic literature review (S.L.R.) is carried out to collect secondary data from scientific journals and development corporations. For primary research, top managers and owners from ten large-size fashion and textile companies were qualitatively interviewed to validate the secondary data. The value chain analysis (V.C.A.) model was used to perform a stage-wise analysis to provides an assessment of the current scenario and recommend solutions accordingly.

Existing literature discusses the impact on the Indian economy in general and there is no significant research on the fashion and textile sector. In accordance with the empirical evidence, the author has developed a digital value chain model that is novel to the sector. It shall help both the domestic and export sector to come back to business and prepare for a similar crisis in the future.

## Introduction

The fast spread of the COVID-19 virus has affected the globe posing enormous health, economic, environmental, and social challenges to its population. This pandemic can be accounted for of the most extreme challenges that mankind has faced in the modern times (Chakraborty & Maity, 2020).

The outbreak has disrupted the majority of the global supply chains across South and Southeast Asia. The economic depression is visible across the globe but emerging nations are suffering the deepest impact.

India has registered the second-highest coronavirus cases in the world^1^ and it presents a new set of opportunities and obstacles to the textile and apparel industry. In developing economies, the social and health care systems are not robust enough to handle the large population, and in the case of such pandemics, the challenge intensifies. The series of lockdowns in the nation has put the textile and apparel industry to a complete standstill. Negative growth patterns, international trade deficits, unemployment, income, poverty, factory shutdowns, retail closures, labor displacement, and shortages are some of the noteworthy challenges that the sector has faced in the current times (Sahoo & Ashwani, 2020; Sen et al., 2020). According to Sahoo and Ashwani (2020), production may decrease from 5.5 to 20%, exports from 13.7 to 20.8%, imports from 17.3 to 25%, and MSME net value added from 2.1 to 5.7 if the current situation continues.

In the initial days of the pandemic, the industry could not have imagined that to face such a long standstill. To worsen this effect, consumer consumption also has been deteriorating sharply for the first time in several decades.

The Indian business development model depends on the export-led-growth (Mishra, 2020), and hence it could experience a massive impact on growth due to the mandatory lockdowns. This paper employs the Value chain analysis (V.C.A.) method to delve particularly into each stage of the value chain and bring out positive and negative aspects of the current crisis. The existing researches (Panigrahi et al., 2020; Sahoo & Ashwani, 2020; Sen et al., 2020) has emphasized the impact of the pandemic on the sector. However, this research goes a step ahead to digitize the value chain and implement the model for future shocks. The author has compiled empirical evidences in academia, research, and industry to deliver a set of managerial solutions to the stakehpolders in the value chain. With this rounded approach in mind, this study shall delve into the following research questions:

RQ1: What are the consequences of COVID-19 on the Indian fashion and textile sector?

RQ2: How can digitization help the sector to fight through the COVID-19 crisis?

The article is organized as follows. In “Theoretical background” section presents the outlook of the sector and current obstacles; "Method" section highlights the methods and "Results" section discusses the results of interviews with the factories. Finally, "Discussion" section and "Further scope of research" section outlines the recommendations and conclusions for the stakeholders in the sector and way forward.

## Theoretical background

### The outlook of Indian apparel sector

India is home to the world’s largest domestic and export textiles and apparel sector. The republic has a history of fine craftsmanship and began exporting in the mid-1960s (Chatterjee & Mohan, 1993). Since then the sector has contributed to exceptional socio-economic progress for the nation in the last four decades. Currently the sector values at US$ 200 billion and has contributed it contributed to India's gross domestic product (3%), industrial manufacturing (13%), export earnings (12%) and provides direct employment to a workforce of around 45 million (Majumdar et al., 2020).

Besides mainstream business, textiles are pertinent to the history and culture of the republic. Mohandas Gandhi encouraged Khadi (home woven cloth) both as a product and a symbol of the swadeshi movement to establish economic independence from the British government (Ghosh, 2009; Trivedi, 2007). Culture is an integral part of the Indian environment and its imprint is visible on fashion and textiles. The geography comprises 28 states and 8 union territories^2^ displaying a kaleidoscope of cultural heritage. Fabrics and surface ornamentation techniques are indigenous to these states and have produced countless meters of intricately woven and printed textiles (see Table 1).

**Table 1:** Fabrics of India

Fabrics	States
Kalamkari, Mangalgiri Fabric	Andhra Pradesh
Banarasi Silk, Chikankari Embroidery	Uttar Pradesh
Sambalpuri Fabric, Bomkai Sari	Odisha
Ikat Fabric	Hyderabad
Chanderi	Madhya Pradesh
Paithani Brocade, Narayan Peth Sari	Maharashtra
Patola Fabric, Bandhni	Gujarat
Pashmina	Kashmir
Phulkari	Punjab
Bandhni, Kota Doria, Ajrakh, Bagru Print, Sangneri Print	Rajasthan
Kanjivaram	Tamil Nadu
Mysore Silk, Ilkal Sari	Karnataka
Muga Silk	Assam
Kasavu	Kerala
Madras Checks (Plaid)	Tamil Nadu
Bhagalpuri Silk	Bihar
Lepcha	Sikkim
Kantha	West Bengal
Kunbi Fabric	Goa

The ministry of Textile (http://texmin.nic.in/.) is responsible for the textile advancements in the country. Majority of the national garments production (80%) is concentrated in ten cities: Kolkata, Mumbai, Tirupur, Ludhiana, Indore, Bellary, Jaipur, Bangalore, Chennai, and Delhi. The textiles and apparel sector are robust across the entire value chain from fiber, yarn, fabric to apparel.

A well-structured textile and garment production ensure strong domestic retail. Young and educated growing Indian middle class makes the biggest markets in the world (Contractor et al., 2015). The retail marketplace is well furnished across fashion segments with both local and foreign brands (Zara, Armani, Forever21, Vero Moda, Calvin Klein, Diesel, or Uniqlo). The nation is going through a digital revolution and has seen a visible rise in online sales (Kaushik & Dhir, 2019).

### The sector vs COVID-19

India is surrounded by garment-producing neighbors and in the last decade and the buyers have constantly shifted to chase lower prices. The Indian garment manufacturers are now coping up with this rising competition as the big box retailers have further started shifting to Africa for even lower prices.

The sector saw a stumbled growth due to slow demand in the western countries and a number of other issues such as technology upgradation and weak infrastructure (Kathuria 2018; Anthony & Joseph, 2014).

Bangladesh has appeared as a clear winner due to lowest labor costs in the South Asian region and this made R.M.G. sector an attractive option over other South Asian countries (Kurpad, 2014). Since then India has been losing its share of the world apparel trade to Vietnam, Bangladesh, China, and Turkey in areas such as apparel, cotton fabric, and carpets; in 2016 it was 3.5%, compared to 6% in 2013 (Ray, 2019). All these factors have weakened the sector in the past and as the COVID-19 crisis struck, it further broke the sector down across the value chain.

The covid-19 crisis affected the Indian fashion and textile sector holistically. Kanupriya (2021) states that the effect of crisis could be understood by examining the demand-side factors (social distancing, consumer demand, and exports) and the supply-side factors (production, supply chain, employment, prices of essential raw materials, and imports). The manufacturing activity across Asia was halted due to canceled orders and unavailability of raw materials. Due to the mandatory lockdown, thousands of garment factories and textile factories (40,000 in Tamil Nadu^3^) had to shut down causing a major disruption in the supply and demand. Apparel export promotion council study reported that, 83% of export orders had been wholly or partially canceled. The apparel export sector suffered a huge hit as the buyers (U.K., U.S.A, E.U.) canceled the order or stopped placing new orders. This led to an immediate inventory buildup and overhead costs at the manufacturer’s end.

Nation-wise lockdown and factory shutdowns had a high financial impact on the weaker sections of the population which mostly are daily wage earners and engaged in the informal economy. (Sharma et al., 2020). According to the U.S.,^4^ thirty percent of India’s export earnings are made from textile and apparel, and the industry employs 38 million. The salary of these workers ranges from Rs.10,000 to 12,000 (US$ 133 to 160 per month), while the living wage, as calculated by the Asia Floor Wage Alliance (AFWA) is Rs.29,323 per month (US$ 386). The nation will have severe consequences for employment as 81% of employment is informal. In March/April 2020 millions of informal workers struggled to return home as the transportation was also cut off to their villages. Social aspects in the value chain have always been neglected in the past (Mani et al., 2016; Mani & Sharma 2015; Mani et al., 2018) and this outbreak has further exposed the susceptibility and lack of social security of these workers who contribute to the splendid accomplishment of the fashion industry (Majumdar et al., 2020).

Moving to the demand side of the sector, there has been a serious fall in the consumer buying pattern during the crisis. The domestic sector of the country comprises designers and small and mid-size clothing brands who are suffering due to shuttered retail stores. The biannual fashion week was canceled by the Design Council of India which further led to weakening the promotion campaigns for the designers. The mandatory social distancing led to the closing of malls nationwide and the retail selling came to a standstill. The local businesses were not prepared to shift the selling online and thus the losses multiplied by the passing time.

## Method

### Data collection

This study begins by assessing secondary data to understand the impact of the pandemic in the sector. A systematic literature review was performed to collect secondary data from scientific databases (Scopus, Web of Science and ScienceDirect); reports from N.G.O.s (Asia Floor Wage Alliance), development organizations (World Bank, I.L.O.), and governmental organizations from India (AEPC, Ministry of Textiles), National Account Statistics, a publication of the ministry of statistics and programme implementation (MOSPI); Handbook of Statistics on Indian Economy and Monthly Bulletin, Reserve Bank of India; Export–Import Database, Ministry of Commerce, Government of India. To minimize bias, explicit and systematic methods were used while reviewing articles and all accessible evidence and, thus providing reliable findings from which deductions can be drawn. (Moher et al., 2009; Snyder, 2019).

To substantiate the secondary data, in-depth qualitative interviews were conducted with 10 company owners and top managers (exporters and domestic brands). Purposeful sampling (Gentles et al., 2015; Kuzel, 1999) was used to select the company as it maximizes the depth and richness of the data to address the research question. The selected companies are large-scale manufacturing houses (over 300 employees) with a presence of over 30 years in the market. In-depth Interviews with both domestic brands and exporters helped to compare and contrast their opinions to ensure the validity of the data (Golafshani, 2003). Open-ended interview guidelines were developed to collect primary information on the problems in the fashion and textile value chain (Creswell et al., 2007). Table 2 shows the information collected and the development of themes from the companies.

**Table 2:** Problems faced by companies

Hindrances	Type of companies
Factory shutdowns and excess inventory	Exporters and domestic brands
Canceled orders and financial stress	Exporters
Rising shipping costs	Exporters
Lack of technological advancements	Exporters
Labor crisis	Exporters and domestic brands
Unsold inventory	Domestic brands
Shifting to online selling	Domestic brands

### Method of analysis

The article is organized according to the value chain analysis (V.C.A.) method which visualizes the problems and provides stage-wise solutions for the sector. The V.C.A. method is applied by authors (Koc & Bozdag, 2017; Khurana & Ataniyazova, 2020) as a tool to visualize the garment value chains across the world. Further on the V.C.A dissects the industry into strategically significant sectors to comprehend its effects and highlights the sources of potential competitive advantage (Faroukhi et al., 2020a; Prajogo et al., 2008).

**Fig. 1:**
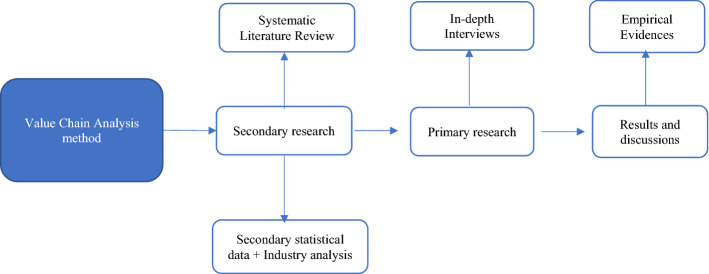
Methodological framework

The methodological framework consists of secondary and primary researches to dig deeper into the current scenario in the Indian value chain (see Fig. 1). With the help of the V.C.A. method, digitization of the value chain is proposed at each stage and the authors show the methods of implementation in the discussion section. As digitization becomes a mandate in the global value chains the Indian sector could take this challenge as a strategy for the future.

## Results

The in-depth interviews with the factory owners and the managers in export houses and brands established the impression of the crisis on the routine working of the sector. While interviewing the factory owners it was found that few buyers (selling online^5^) are currently placing orders to large/established vendors but have shrunk their order quantities, fixed (low) prices, increased quality standards, and demand multiple styles. For the manufacturers, it means a lot of time-consuming product development for smaller orders. This stands against the economic order quantity model (Schwarz, 2008) and thus lowers productivity and profits. Due to the uncertain demand and fragile trends, the buyers became much more apprehensive about on-time deliveries and demand air deliveries (DHL/FedEx). The shipping freight which used to cost 5 USD/kg has more than doubled to 13.5 USD/kg. Thus, the logistics are at a historical high and have increased the unit cost of a garment. Further on, all these aspects have impacted the cash cycle at the manufacture's end as the buyer sells the merchandise and pays back. The credit limit which used to 60 days has increased to 120 days making higher debts with the banks. As the payment cycle requires double the time, the vendors have to pay back almost double the interest on the loans. These circumstances have finally decreased the margins steeply in the export sector, in many cases, the vendors can’t reach break-even. The pandemic has taken its maximum toll on the workers as millions lost their jobs and there’s more ahead. During the interviews, the factory owners confirmed that there is no certainty of business and they will have to lay off staff as the cases are rising in the country. Due to large numbers, the workers have received very scanty financial and health assistance from the government and industry. The larger the nation, the deeper is the impact of the crisis on its economic stability.

In the in-depth interviews, it was realized that despite the technological advances in the garment sector not much had been put to practice. Matt & Rauch (2020) indicate that, most advanced companies that have performed digitization of their activities and workflows, are still not entirely equipped to face the challenges of the digital transformation. Some of the garment factories had the technology but the staff was not trained or used conventional methods of working. For the factory owners, this meant an investment in terms of money and human effort. They also feared the fact that automating the processes could eliminate jobs which could intensify the unemployment crisis shaped by the epidemic. Due to these reasons, there could be struggle to the introduction of technologies in the Indian sector. Moreover, this slow adaptation is also one of the reasons which have delayed the progress of the sector against China.

Coming to the domestic brands, the struggle is not less either. Social distancing measures prohibit entry to malls and market places, reduced public movement has created a huge void in the retail spaces in the country. The managers confirmed that as the buying volume of the consumers dropped sharply in the last 6 months it has serious effects on the local economy. Stores inside malls have stopped paying rents or have vacated as they were closed until June 2020 in major cities of the county. As the government released the lockdown the sales staff returned to their jobs However, the stores saw no substantial footfall. Most of the domestic brand owners suffer from unsold inventory/deadstock, overhead costs, and shall result in staff reduction as the turnovers have dropped intensively. The cash flow cycle is disturbed as a major portion of the money is blocked into finished products waiting to be sold. As digital marketing is taking the main stage for selling and promoting around the world during the pandemic, the local brand owners and designers were asked if they were familiar with digital marketing. It was observed that a majority of them were aware of the omnichannel strategy (Lorenzo-Romero et al., 2020) but implementation was still a difficult task for them as they had no significant training in this area. This had led to a limited online selling revenue to the local brands. This indicates that there is a large gap in the digitization of the value chain which has led to a steep financial crisis.

## Discussion

The current crisis has enforced a “refresh” moment in the garment sector. The period and aftermath of the pandemic were never anticipated by the stakeholders. However, as change (*new normal*) is essential for each industry, it is time to handle the crisis with a set of managerial solutions so it revives back. Keeping in mind the size of the sector, this section provides a set of recommendations that are specifically tailored for the Indian environment.

### Strengthening the domestic sector

The Indian sector is an example of a comprehensive value chain. From raw material to the final consumer, the economy supports it all. Big box retailers were better at designing fashion merchandise and also had higher social status and hereby, they quickly gained the market share in the last decade. Foreign brands occupied the major market places in big cities and the national brands have to move to smaller cities to sustain the business. Kinra (2006) state that consumers tend to have positive brand image perceptions towards international brands as the major weakness of Indian local brands was their inferior degree of social status. The Indian government has always emphasized national products through the Make in India (2014) campaigns. These initiatives were launched to boost the entrepreneur in India and stand against foreign brands and imports. A good example is a current shift on the market; during the pandemic, Indian manufacturers have completely stopped importing products from China. Around 118 Chinese applications such as TikTok, Shareit, etc. were also banned due to political tension. This movement has stirred the consumer sentiment towards the local brands across industries but the companies have to strive to make up trendy and innovative fashion merchandise to gather market share. The Indian consumer market is quite huge, with over a billion people and if served with the right merchandise the sector could achieve a standard growth in the desired time. As soon as the local fashion merchandise gains acceptance in the market the whole value chain shall start to recover from the crisis and would not depend on imports for the future.

### The digital business modeling

The pandemic has fast-tracked the need for digitization across the value chain. On one end where the companies have suffered working offline, the digital technology and platform economy firms (Amazon, Alibaba, Google, and Netflix) continue to grow in importance and are moving to the center stage in organizing key infrastructure (Klein, 2020). Pandey & Pal (2020) suggest that digital transformation technologies should be implemented by companies as part of innovation strategies. While banking, education, and health care sectors are quickly adapting to the digital changes, the fashion and textile sector is still lacking behind.

**Fig. 2:**
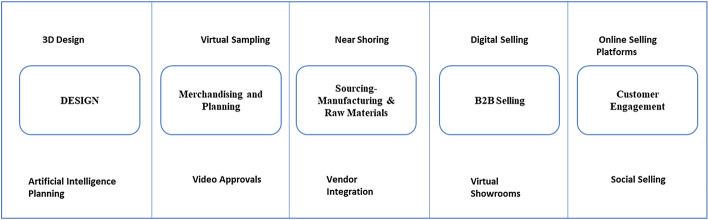
Value chain digitization

The traditional approaches of selling fashion have been disrupted by digital technologies as they become an integral part of the industry (Sun & Zhao, 2018). Digitizing the manufacturing process could decrease the lead time, lower manufacturing cost, lessen periodical maintenance by predictive maintenance, minimize machine breakdown time, and create a synergistic setting of production with zero re-work (Tareque & Islam 2020). To digitize the process, the author developed a business model (Fig. 2) that digitizes each stage with optimized financial investments. Fashion product development is complex as it requires several stages and a strong integration within the supply chain (Ha-Brookshire, 2017). Starting from the design stage, computer-aided design has been quite beneficial in decreasing the lead time. However, this process is 2D and needs to be transformed into a 3D pattern generation. Kim and Park (2007) divided a garment into two zones, fit and fashion. The *fit zone* digitizes the body scan data so that it can provide optimum fit as well as the ideal silhouette of the garment. The *fashion zone* determines the aesthetic appearance of the garment that users can design garments with various silhouettes instinctively. To ease the design process artificial intelligence or machine learning, can predict fashion trends with greater precision and swiftness through the collection of user and market data. Virtual models of products based on generative design algorithm could also be developed (Sun & Zhao, 2018).

Merchandising and planning are critical and time-consuming stages of the value chain. 3D visualization of drape and fit on virtual model or avatar shall eliminate the exhaustive process of physical sample generation, less traveling expense decreasing CO2 emissions, and minimize fabric waste (Lee & Park, 2017; Jhanji, 2018; Hwang Shin & Lee, 2020), leading to a sustainable production. As online selling remains the preferable way of commerce in the country, virtual try-on shall ensure garment fitting and speed up buying decisions (Liu et al., 2017; Song & Ashdown, 2015). Numerous virtual try-on programs, such as Clo 3D, Lectra 3D Prototype, OptiTex, and V-Stitcher 3D could be fused into the value chains (Sayem et al., 2010).

The Indian manufacturers were importing raw materials and finished products from the neighboring countries and the majority was from China. As the borders were closed it disrupted the whole value chain. The factories have no raw materials or the stores were out of inventory. The fashion industry is characterized by high unpredictability, low sales probability, varying consumer demands, and fast trends (Brusset & Teller, 2017). In view of the current crisis, the author highly recommends using the nearshoring/back shoring strategy for sourcing (Kinkel, 2012; Fratocchi et al., 2014; Martinez-Mora & Merino, 2014). Raw materials and finished products sourcing has been an integral part of the final profits of the value chain and low-cost countries are seen as an opportunity to profit from cost advantages (Baraldi et al., 2018; Tate & Bals, 2017, Macchion & Fornasiero, 2020). In the Indian scenario, a lot of production moved out to the neighbors as the companies wanted to achieve higher profit margins. During the pandemic, the small and medium scale vendors suffered the most as they had no orders and had to close down immediately. Nevertheless, now is the right moment to integrate the local vendors which will profit the whole value chain and keep the cash flow cycle secure inside the nation. The large manufacturer should integrate with the local suppliers through enhanced I.T. capabilities (Liu et al., 2013, 2016), supplier relationship management capabilities (Wagner et al., 2018), supply chain integration (Huo et al., 2018; Li et al., 2016). This integral integration shall help the money to circulate in the economy internally and stabilize for cash crunches. Further on, for selling merchandise in B2B, virtual showrooms and digital selling will be very supportive methods. Indian manufacturers should transform into virtual offices where the buyers can select the merchandise through visual representation techniques.

Towards the end of the value chain, the Indian consumer shall hold the most important in times of crisis. Due to a large population, it can be believed that if the companies targeted the national consumer with the optimum merchandise, the crisis can be handled. There are 4000 cities and towns in India and buying fashion online is still not a common practice in the smaller towns. Presently, only Amazon has been able to establish the largest logistic network for general products in the country. This represents a large opportunity for new online selling portals which could have local suppliers and take advantage of untapped consumers. The companies should further look into easing the consumer decision process to speed up the sales (Kim, 2020).

Social media influencing and online portals are a natural fit for selling fashion. Social media may not be considered useful unless the interactions taking place on social media forums result in online purchases (Das & Mandal, 2016). The social media trend not only is a memorable shopping experience but also is impactful in an aesthetic experience and even enhances our sensory perception (Silvestri, 2020). India has a large young population which has represented a much faster ease of accepting online sales and social media. Shen et al. (2017) prove that when the impacts of social influences are larger, the supply chain should provide a better online retail service across fashion segments. Hence, the Indian value chain should work in direction of strengthening its presence in the sub-continent.

In summary, digital transformation does not necessarily mean that Indian companies must abandon their current business models. On the contrary, the new digital value chain model shall complement the existing traditional ones and give them a futuristic vision. India has a competitive advantage over other emerging economies in terms of technological capabilities. Therefore, it is recommended to digitize the value chain to combat the crisis.

### Dealing with the labor crisis

India officially records 63 million micro-enterprises, employing 107 million people (Government of India, 2020). The unregistered manufacturing units, daily-wage workforces, and small traders account for a further 200–300 million workers. As mentioned in Sect. The sector vs COVID-19, the laborers have suffered most during this crisis. The garment companies along with the Ministry of Textile should start thinking about how to deal with the immediate and long-term consequences of the pandemic on the labor class. Blustein et al., (2020) argue that it would be vital to cautiously analyze external circumstances of the jobless individuals, including the prospect of re-employment, monetary condition, family structure, and living circumstances. It should be considered that laying off the workers is not a viable solution as after the pandemic it might be very tough to find skilled workers. Khurana (2018) argues that skilling the human resource has been instrumental in success for emerging economies. However, if the daily wage workers fail to find employment in this sector they tend to change the industry to sustain their livelihoods. Social compliance standards have been a pressing issue in the Indian environment for a long time, and at this crisis time, it needs attention.

In a very short while, the digital transformation shall be the new mandate. To succeed in this new transition, educated managers and human resources will play a key role. The companies should assess the individual's strengths and growth edges and train them further in the desired area. The success of the digital business model (Fig. 2) depends on the way it shall be implemented through the value chain. Almeida et al., (2020) argue that the accomplishment of digital economy rests on on a public and private approach for the digitalization of education and training of the whole population in information and communication technologies. Training of 3-d development software, virtual sampling, and fitting, online marketing and selling, managing the logistics online, etc. are some of the areas where the human resource should be trained to make the process seamless. This shall increase the global credibility of the sector as the exporters shall be able to process the orders faster and efficiently.

### Financial modelling

In the interviews conducted it was found that the cash crunch in both the domestic and export sectors. The owners are worried about the increasing interest rates and lack of financial trust from international buyers. The last decade has been quite tricky for the financial institution in the nation. In the last 5 years, the government implemented new strategies such as demonetization (Rajagopalan, 2020), new tax policies such as G.S.T. (goods and services tax) (Mukherjee, 2020). These shocks have created a lot of mistrust in the business environment. The banks are now very mindful of extending a helping hand to the manufacturers and this has caused a major halt in the operations of the sector. In this period, the ministry of Textiles shall play a very decisive role in supporting the sector and especially the S.M.E.’s. Authors (Kolev, 2016; Brahmana et al. 2021; Konara and Ganotakis, 2020) recommend a diversification strategy through which the firms can achieve funding by selling their subsidiaries. Such a strategy not only a generates extra cash flow but also transforms the organization in a competitive and planned way.

By applying such a strategy, the large companies could fragment themselves reducing risks and cash crunches. Moreover, the S.M.E.’s can financially integrate to strengthen the whole value chain. A set of special policies regarding financial stabilities should be issued for the fashion and textile companies to provide short-term support. Panigrahi et al (2020) state that under the TUFS (Technology Upgradation Fund Scheme), Reserve Bank of India decided to reduce the rate and introduce a subsidy scheme up to 1–2%. This will help them recover from the slump and bring the operations back to normal.

### Sustainability vs economic reforms

On a positive note, the Covid-19 crisis served as a disruption for the Indian production and consumption patterns which was the need of the hour. This disruption has helped the nation to slow down production and consumption which was the need of the hour. The global fashion value chain is under the sustainability scanner for over two decades due to its social and environmental cost (Khurana and Ricchetti, 2016; Niinimäki et al., 2020; Khurana & Muthu, 2022).

The nation contributes significantly to population (18%) and territorial air pollution (26%) of the globe (Balakrishnan et al., 2019). Overpopulation and excessive consumption patterns resulted in making New Delhi and Mumbai one of the most polluted and dirty cities in the world. However, in the lockdown period, these cities have noted a considerable reduction in air pollution, energy consumption, and transportation (Shehzad et al., 2020). Energy consumption in India also declined during March 2020 and the lock-downs helped in improving air quality in India (Shehzad et al., 2020). Further on, there is a notable decrease (500%) in sewage and industrial wastes (Singhal & Matto, 2020). Moreover, India's river, Ganga has seen the improvement in the water quality for three decades. Subsequently, reduced consumption aided a significant drop in waste generation and a major change was observed in the landfills/dumpsites in the last two months (Somani et al., 2020).

However, in the current scenario, it is evident that the companies are quite eager to cover up for the losses in the year 2020. Within no time, the degradation of the environment shall be similar as it was in the past. As much as it is required to continue this economic progress, it will be very essential to maintain a balance. This disruption has given the stakeholders a chance to think of revival in conjunction with people, planet, and profits.

### Further scope of research

This work has delved into upgrading the fashion and textile sector in India and hence, it posits some further research thoughts. First, at the broadest macroeconomic level, the question opens up significant space for supply chain monitoring and production systems to be re-engineered over the next financial quarter and whether to choose global or local sourcing. Second, the adaptation and implementation of digitization in the value chain over time. There is substantial work in the area to be done in large, medium, and small-scale industries. Third, post-crisis will be very essential to monitor the environmental and social impact of the fashion and garment sector of the economy. It will be very crucial to stabilize and bring back the labor force to the new normal. Lastly, what strategies should the sector imply to be ready for future shocks as this is not the last unprecedented event in world economic history.

## Conclusions

The COVID-19 crisis took much longer than expected and has particularly hit hard the developing countries (Ahmed et al., 2020; Sumner et al., 2020). The objective of this research is not only to support the Indian sector but also to be an inspiration for other emerging economies that are suffering a similar set of hindrances. Digitization is the future of all industries today and the fashion and textile sector should start to think intensively about it. The garment industry is of economic importance to India and needs attention from its stakeholders for a robust future. In this work, the author has tried to present a holistic view of the sector during and post COVID-19 times. A comprehensive value chain analysis envisioned the current hindrances and provide feasible solutions. The current crisis is the time to realize that challenges can indeed be transformed into opportunities. The digital transformation was quite in progress in the Indian economy and this crisis led to a whole new revolution. In particular, this study contributes to this issue by providing a novel digital value chain model. The digital model shall be very instrumental in stabilizing the value chain as we still can`t figure out the time span of the crisis. It will further help the Indian sector sustainably revive and make a place for itself in world trade.

## Data Availability

The datasets used and/or analyzed during the current study are available from the corresponding author on reasonable request.

## Footnotes

1 https://www.worldometers.info/coronavirus/country/india/.

2 https://knowindia.gov.in/states-uts/

3 https://www.theglobeandmail.com/business/international-business/asia-pacific-business/article-indias-coronavirus-lockdown-leaves-garment-workers-stranded-in/.

4 https://www.usitc.gov/publications/332/PUB3401.pdf.

5 Selling online has a higher rate of returns as compared to physical retailing and thus higher quality is expected from the vendor. Also, every day discount method offers lower unit prices to the manufacturers.
